# Highly efficient gold(I)-catalyzed Overman rearrangement in water

**DOI:** 10.3762/bjoc.7.88

**Published:** 2011-06-08

**Authors:** Dong Xing, Dan Yang

**Affiliations:** 1 Department of Chemistry, The University of Hong Kong, Pokfulam Road, Hong Kong, China

**Keywords:** allylic trichloroacetamides, allylic trichloroacetimidate, gold(I) chloride, Overman rearrangement, water

## Abstract

A highly efficient gold(I)-catalyzed Overman rearrangement of allylic trichloroacetimidates to allylic trichloroacetamides in water is reported. With this environmentally benign and scalable protocol, a series of C3-alkyl substituted allylic trichloroacetamides were synthesized in good to high yields.

## Introduction

The aza-Claisen rearrangement of allylic trichloroacetimidates to allylic trichloroacetamides (Overman rearrangement) is a powerful and attractive strategy for the synthesis of allylic amines from readily available allylic alcohols [1–2]. This transformation can be conducted thermally at high temperatures or by transition metal catalysis under very mild conditions [3–7]. Asymmetric induction has been achieved with certain types of transition metal catalysts (e.g., palladium complexes) in combination with chiral ancillary ligands [8–13]. However, although a large number of late transition metal catalysts have been used for different types of [3,3]-sigmatropic rearrangements [14–15], only Pd(II) and Hg(II) salts have found wide application in Overman rearrangements. In recent years, gold catalysts have been successfully applied to a series of [3,3]-sigmatropic rearrangements, such as the rearrangement of propargylic esters to allenyl esters [16–21], allenyl carbinol esters to 1,3-butadien-2-ol esters [22] and the isomerization of allylic acetates [23–24]. However, when they were used as catalysts for the Overman rearrangement, the substrate scope was limited and only poor to moderate yields were achieved [25–28]. Very recently, our group developed an efficient gold(I)-catalyzed decarboxylative aza-Claisen rearrangement of allylic *N*-tosylcarbamates for the synthesis of *N*-tosyl allylic amines [29]. This reaction was performed in water and therefore represented an environmentally benign protocol [30–34]. We decided to apply this extremely mild catalytic system to the Overman rearrangement of allylic trichloroacetimidates.

## Results and Discussion

Trichloroacetimidate **1a** was prepared by the DBU-catalyzed addition of *trans*-2-penten-1-ol to trichloroacetonitrile [10,35]. With this substrate, the catalytic activities of different gold(I) complexes in H_2_O were examined. When **1a** was subjected to the optimal catalytic conditions previously reported by our group (5 mol % AuCl/AgOTf at 75 °C) [29], the desired allylic trichloroacetamide **2a** was obtained in 91% yield in a reaction time of 1 h (Table 1, entry 1). Gold(I) complexes with phosphine ligands, Au(PPh_3_)Cl or Au[P(*t*-Bu)_2_(*o*-Ph)Ph]Cl, in place of AuCl gave none of the desired product (Table 1, entries 2 and 3). Further screening revealed that AuCl alone could catalyze this reaction with high efficiency to give **2a** in 92% yield in 2 h (Table 1, entry 4). On the other hand, with only AgOTf as the catalyst, the formation of **2a** was not observed and substrate **1a** decomposed completely (Table 1, entry 5). In the absence of AuCl, substrate **1a** remained unreacted, even when the temperature was increased to 100 °C for 3 h (Table 1, entry 6), indicating that the gold(I) catalyst is indispensible for this transformation. This gold(I)-catalyzed reaction could be performed at room temperature, albeit with a prolonged reaction time (Table 1, entry 7). When the temperature was raised to 55 °C the reaction was complete within 2 h and in excellent yield (94%; Table 1, entry 8).

**Table 1:** Optimization of reaction conditions.^a^

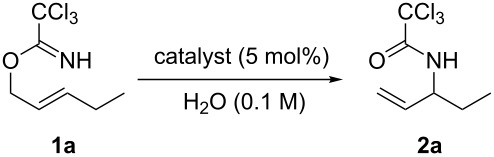				

Entry	Catalyst (mol %)	Temp (°C)	Time (h)	Yield (%)^b^

1	AuCl/AgOTf	75	2	91
2	AuPPh_3_Cl/AgOTf	75	12	<5
3	Au[P(*t*-Bu)_2_(*o*-Ph)Ph]Cl/AgOTf	75	12	<5
4	AuCl	75	2	92
5	AgOTf	75	12	<5
6	–	100	3	<5
7	AuCl	rt	12	90
8	AuCl	55	2	94

^a^Reaction conditions: 0.3 mmol of substrate, 5 mol % of the catalyst, 3 mL H_2_O; ^b^yield determined by ^1^H NMR with nitrobenzene as internal standard.

With the optimized reaction conditions in hand, the substrate scope of this gold(I)-catalyzed Overman rearrangement was surveyed. Different alkyl substituents at the C1 position of allylic trichloroacetimidates, including methyl (**1b**), ethyl (**1a**), *n*-propyl (**1c**) and phenethyl (**1d**) groups, underwent the desired transformation smoothly to afford the corresponding C3-alkyl substituted allylic trichloroacetamides in high yields (Table 2, entries 1–4). The C1-diethylmethyl substituted substrate (**1e**) also underwent the desired rearrangement, affording the desired product (**2e**) in 67% isolated yield (Table 2, entry 5). However, neither the substrates with phenyl (**1f**) nor dimethyl (**1g**) substituents at the C1 position gave the rearranged product (Table 2, entries 6 and 7), indicating that both electronic and steric effects at the C1 position play roles in the rearrangement. Although the substrate scope is currently limited to C1-alkyl substituted trichloroacetimidates, this method is still very convenient and attractive for the preparation of synthetically useful allylic amines. For example, substrates containing either TBDMS- or THP-protected hydroxy groups (**1h** and **1i**) efficiently underwent the desired rearrangement to afford the corresponding products, which are precursors for the synthesis of a variety of β-substituted β-amino alcohols (Table 2, entries 8 and 9). Compound **2j** was also obtained in 71% yield under the reaction conditions from the corresponding trichloroacetimidate **1j** (Table 2, entry 10). Trichloroacetamide **2j** could be transformed to vigabatrin, a GABA aminotransaminase inhibitor [36], in one single step [37].

**Table 2:** Gold(I)-catalyzed Overman rearrangement in H_2_O.^a^

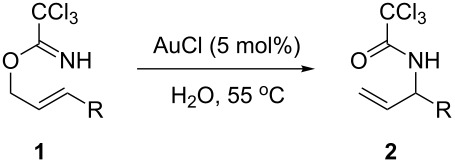				

Entry	Substrate	Product	Time (h)	Yield (%)^b^

1	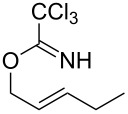 **1a**	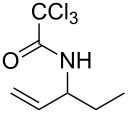 **2a**	2	92
2	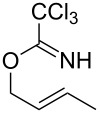 **1b**	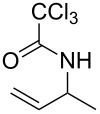 **2b**	2	96
3	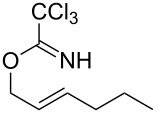 **1c**	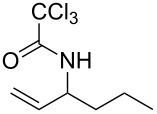 **2c**	2	95
4	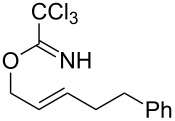 **1d**	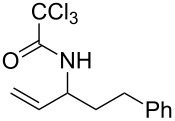 **2d**	2	90
5	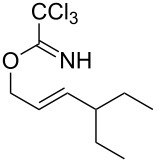 **1e**	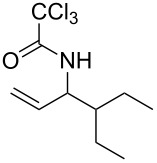 **2e**	3	67^c^
6	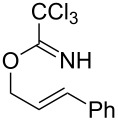 **1f**	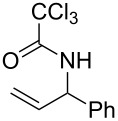 **2f**	3	n.d.^d^
7	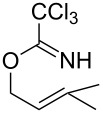 **1g**	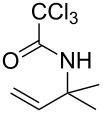 **2g**	3	n.d.^d^
8	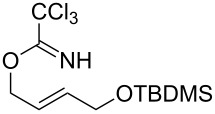 **1h**	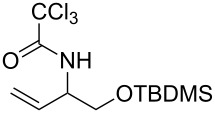 **2h**	3	86
9	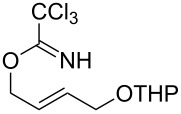 **1i**	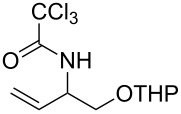 **2i**	2	79
10	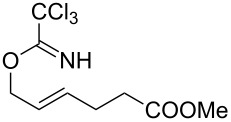 **1j**	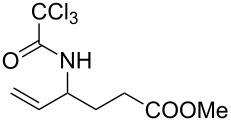 **2j**	6	71^c^

^a^Unless otherwise indicated, all reactions were carried out on a 0.5 mmol scale with 5 mol % of AuCl in 5 mL H_2_O at 55 °C for the indicated time; ^b^unless otherwise indicated, crude yields were reported with >95% purities as determined by ^1^H NMR; ^c^isolated yield after flash chromatography; ^d^n.d. = not detected.

**Scheme 1:**
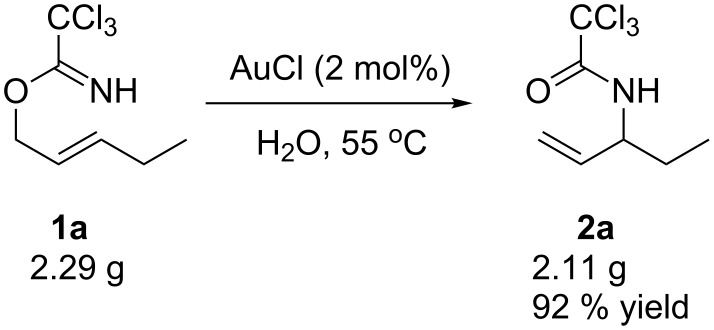
Gram-scale synthesis of **2a**.

One of the most remarkable features of this gold(I)-catalyzed Overman rearrangement is that it is performed in water under very mild reaction conditions. Moreover, this method is extremely clean. After completion of the reaction, simple extraction gave the desired product in high purity, and no further purification step was required. To illustrate the potential utility of this method for industrial applications, a gram-scale synthesis of **2a** was performed with 2 mol % of AuCl in H_2_O (Scheme 1). After reacting at 55 °C for 4 h, the desired product was obtained in 92% yield.

## Conclusion

In summary, we have developed an efficient gold(I)-catalyzed Overman rearrangement for the synthesis of a series of C3-alkyl substituted allylic trichloroacetamides. This transformation was performed in water under very mild reaction conditions and could be carried out on the gram-scale with low catalyst loading and simple work-up procedure, making it potentially applicable to the industrial community for large-scale synthesis. Further exploration of the substrate scope and the development of an asymmetric version of this transformation are currently underway in our group.

## Experimental

### 

#### Typical procedure: Synthesis of 2,2,2-trichloro-N-(pent-1-en-3-yl)acetamide (2a)

AuCl (5.8 mg, 0.025 mmol) was added to a solution of (*E*)-pent-2-enyl 2,2,2-trichloroacetimidate (**1a**) (115 mg, 0.5 mmol) in H_2_O (5 mL) with vigorously stirring in a 25 mL reaction tube. The reaction mixture was heated at 55 °C for 2 h, then cooled to room temperature, diluted with H_2_O and extracted with CH_2_Cl_2_ three times. The combined organic layers were dried over MgSO_4_, filtered through a short pad of celite and concentrated in vacuo to provide 107 mg of 2,2,2-trichloro-*N*-(pent-1-en-3-yl)acetamide (**2a**) (>95% purity as determined by ^1^H NMR). Products **2a**–**2d, 2h** and **2j** are known compounds and their data were identical to those reported in the literature.

## Supporting Information

File 1 ^1^H NMR data and NMR spectra of products **2a**–**2d**, **2g**–**2i**.
