# Supplementary material for: Highly efficient gold(I)-catalyzed Overman rearrangement in water
Source: Beilstein J Org Chem. 2011 Jun 8;7:781–5. doi: 10.3762/bjoc.7.88 (PMC3135174; doi:10.3762/bjoc.7.88)
Supplement: File 1 — 1H NMR data and NMR spectra of products 2a–2d, 2g–2i. [file Beilstein_J_Org_Chem-07-781-s001.pdf]

Supporting Information

for

Highly efficient gold(I)-catalyzed Overman rearrangement in water

Dong Xing and Dan Yang*

Address: Department of Chemistry, The University of Hong Kong, Pokfulam Road, Hong Kong, China

Email: Dan Yang* - yangdan@hku.hk, Dong Xing - xingdong@hku.hk

* Corresponding author

^{1}H NMR data and NMR spectra of products 2a–2d, 2g–2i.

Table of contents

^{1}H NMR data of the product S2–S3

NMR spectra of the product S4–S8

^{1}H NMR data of the products

Compound **2a** [1]: ^{1}H NMR (400 MHz, $CDCl_3$) δ 6.62 (br, 1H), 5.77 (ddd, *J* = 17.1, 10.4, 5.7 Hz, 1H), 5.22–5.15 (m, 2H), 4.35–4.28 (m, 1H), 1.69–1.59 (m, 2H), 0.94 (t, *J* = 7.4 Hz, 3H).

Compound **2b** [1]: ^{1}H NMR (300 MHz, $CDCl_3$) δ 6.60 (br, 1H), 5.85 (ddd, *J* = 17.2, 10.4, 5.1 Hz, 1H), 5.26–5.15 (m, 2H), 4.53–4.50 (m, 1H), 1.34 (d, *J* = 6.8 Hz, 3H).

Compound **2c** [1]: ^{1}H NMR (300 MHz, $CDCl_3$) δ 6.57 (br, 1H), 5.78 (ddd, *J* = 17.0, 10.4, 5.6 Hz, 1H), 5.21 (d, *J* = 17.6 Hz, 1H), 5.17 (d, *J* = 10.5 Hz, 1H), 4.45–4.34 (m, 1H), 1.67–1.51 (m, 2H), 1.44–1.30 (m, 2H), 0.93 (t, *J* = 7.3 Hz, 3H).

Compound **2d** [1]: ^{1}H NMR (300 MHz, $CDCl_3$) δ 7.32–7.17 (m, 5H), 6.56 (br, 1H), 5.84 (ddd, *J* = 16.3, 10.4, 5.6 Hz, 1H), 5.29–5.23 (m, 2H), 4.52–4.45 (m, 1H), 2.71 (t, *J* = 7.7 Hz, 2H), 2.04–1.94 (m, 2H).

Compound **2e**: a light yellow liquid; ^{1}H NMR (400 MHz, $CDCl_3$) δ 6.58 (br, 1H), 5.78 (ddd, *J* = 17.4, 10.3, 5.5 Hz, 1H), 5.23–5.18 (m, 2H), 4.55–4.51 (m, 1H) 1.47–1.38 (m, 3H), 1.36–1.21 (m, 2H), 0.96–0.91 (m, 6H); ^{13}C NMR (100 MHz, $CDCl_3$) 161.2, 135.7, 116.3, 55.1, 45.1, 22.4, 21.8, 11.6; IR (CH_2Cl_2): 3425, 2970, 1713, 1512, 1265 cm^{-1}; LRMS (EI, 20 eV) *m*/*z* 236 (M^+ – Cl, 2), 202 (25), 166 (100), 132 (19); HRMS (EI) calcd for $C_{10}H_{16}Cl_2NO$ (M^+ – Cl) 236.0603, found 236.0604.

Compound **2h** [1]: ^{1}H NMR (400 MHz, $CDCl_3$) δ 7.18 (br, 1H), 5.85 (ddd, *J* = 16.9, 10.4, 5.7 Hz, 1H), 5.28 (d, *J* = 18.8 Hz, 1H), 5.24 (d, *J* = 10.8 Hz, 1H), 4.47–4.43 (m, 1H), 3.83–3.70 (m, 2H), 0.89 (s, 9H), 0.06 (s, 6H).

Compound **2i**: a colorless liquid; ^{1}H NMR (400 MHz, $CDCl_3$) δ 7.16 (br, 1H), 5.87

(ddd, *J* = 17.2, 10.5, 5.2 Hz, 1H), 5.35–5.30 (m, 2H), 4.87 (s, 1H), 4.56–4.51 (m, 1H), 4.01–3.98 (m, 1H), 3.92–3.77 (m, 2H), 3.53–3.50 (m, 1H), 3.39–3.37 (m, 1H), 2.34 (s, 1H), 1.87–1.78 (m, 2H), 1.52–1.50 (m, 2H); ^{13}C NMR (100 MHz, $CDCl_3$) δ 161.7, 133.5, 117.8, 94.5, 63.9, 63.7, 54.8, 31.9, 25.2, 20.3; IR (CH_2Cl_2): 3055, 2986, 1713, 1504, 1427, 1265 cm^{-1}; LRMS (EI, 20 eV) *m*/*z* 199 (M^+ – CH_2OTHP, 69), 166 (100), 132 (81), 71 (35); HRMS (EI) calcd for $C_5H_5Cl_3NO$ (M^+ – CH_2OTHP) 199.9431, found 199.9435.

Compound **2j** [1]: ^{1}H NMR (400 MHz, $CDCl_3$) δ 7.04 (br, 1H), 5.78 (ddd, *J* = 17.0, 10.4, 5.6 Hz, 1H), 5.26 (d, *J* = 16.6 Hz, 1H), 5.22 (d, *J* = 10.2 Hz, 1H), 4.48–4.41 (m, 1H), 3.68 (s, 3H), 2.52–2.36 (m, 2H), 2.07–1.92 (m, 2H).

References

1. Anderson, C. E., Overman, L. E. *J. Am. Chem. Soc.* **2003**, *125*, 12412–12413. doi:10.1021/ja037086r

NMR spectra of the products

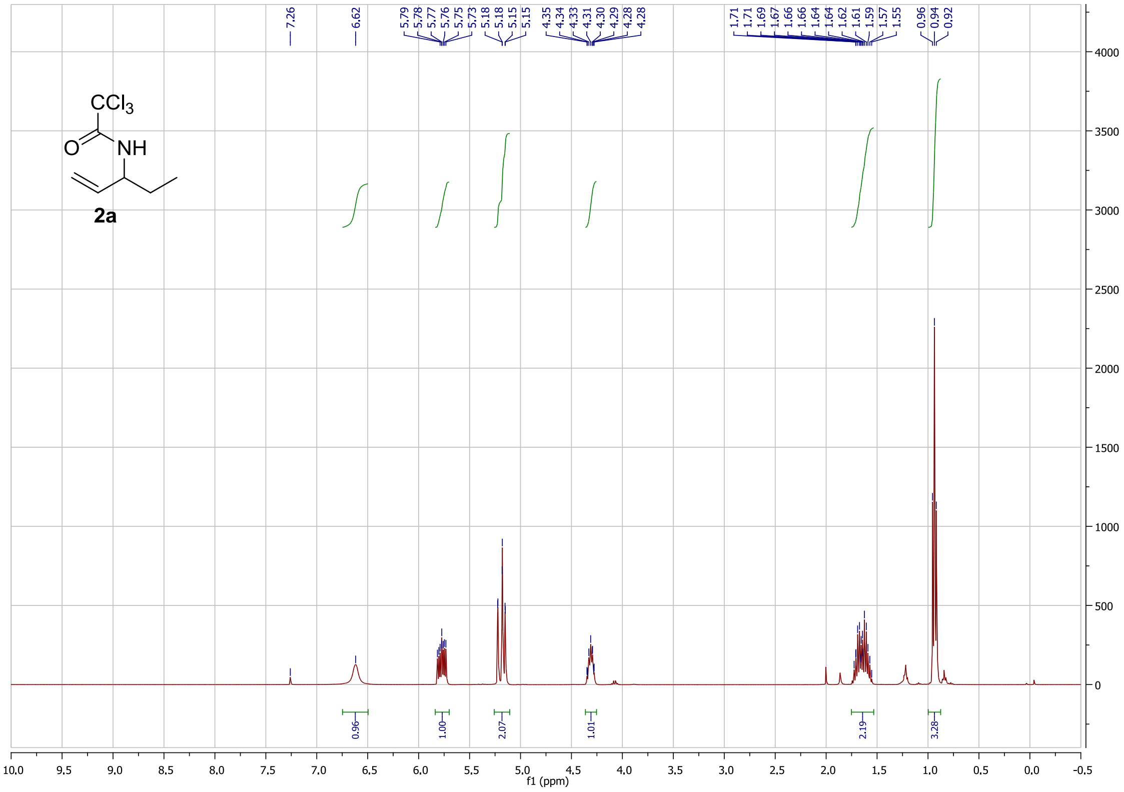

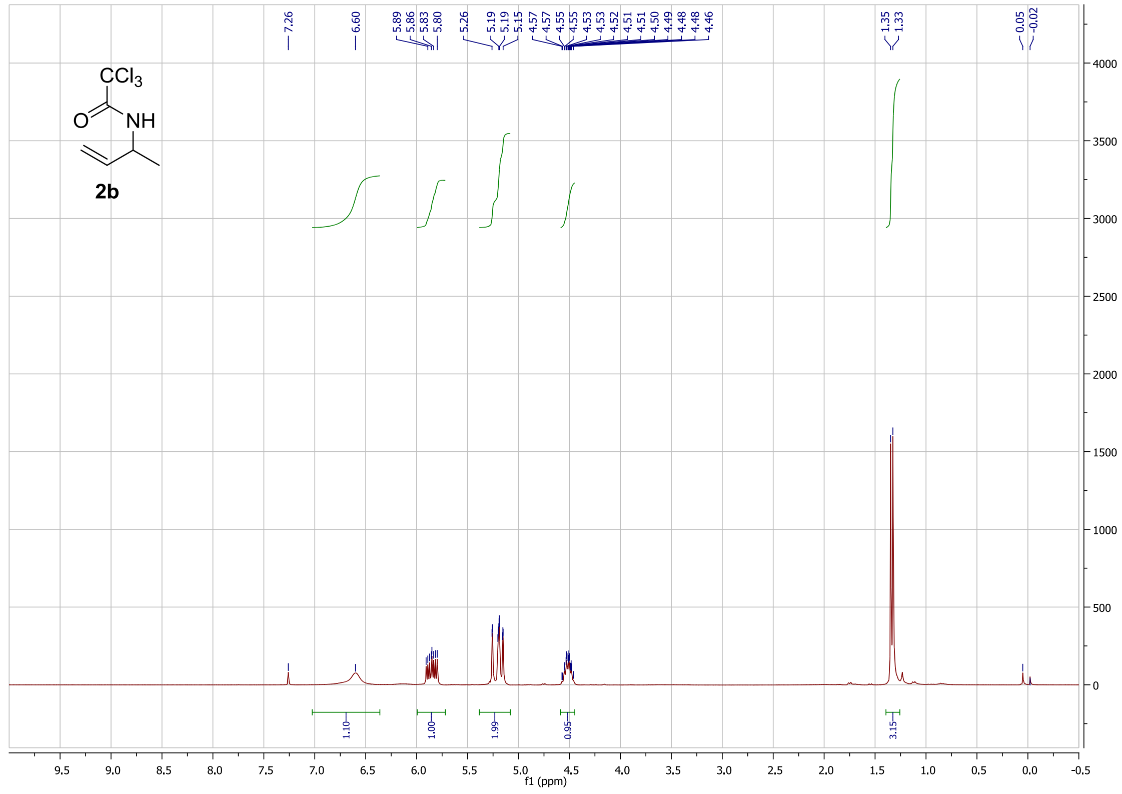

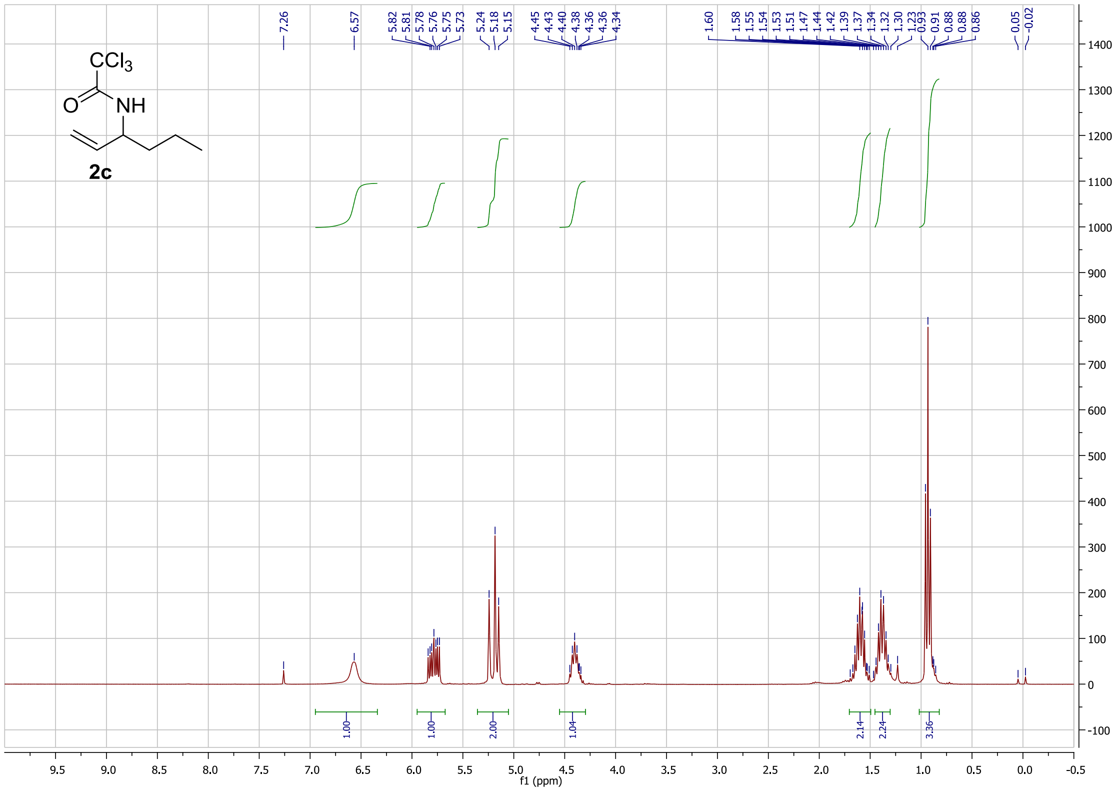
CCl3
O
NH
2c
f1 (ppm)

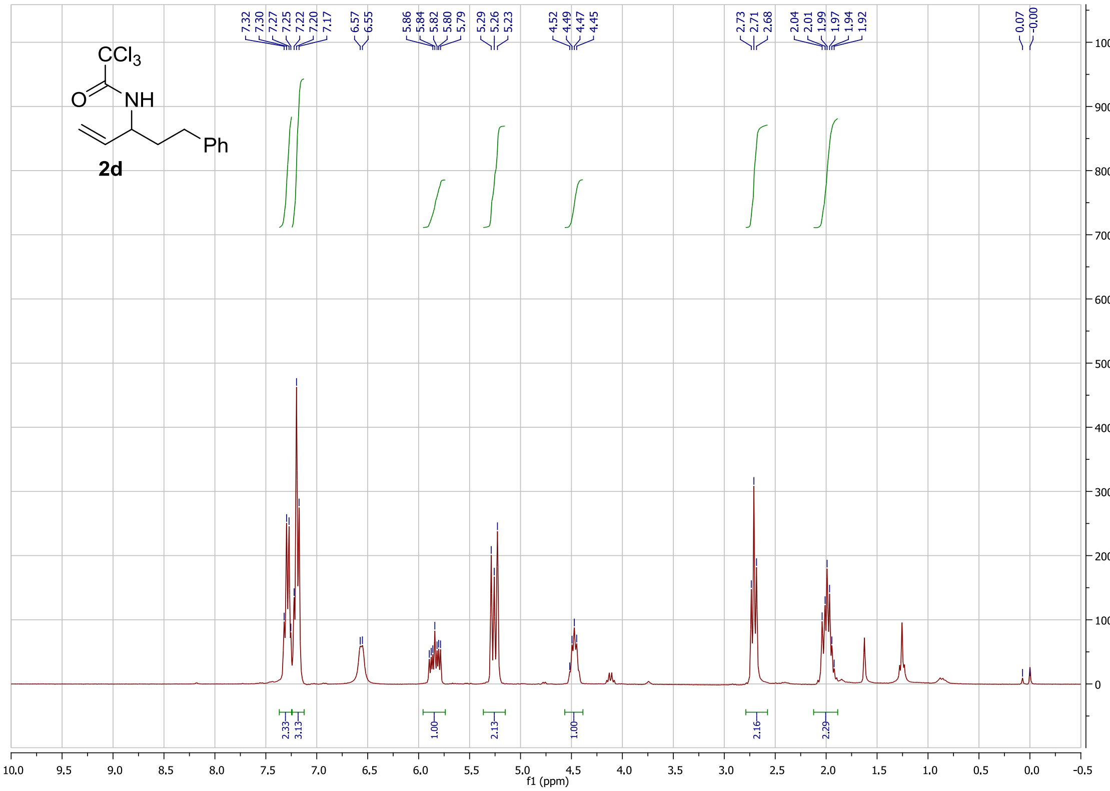
CCl3
O
NH
Ph
2d
f1 (ppm)

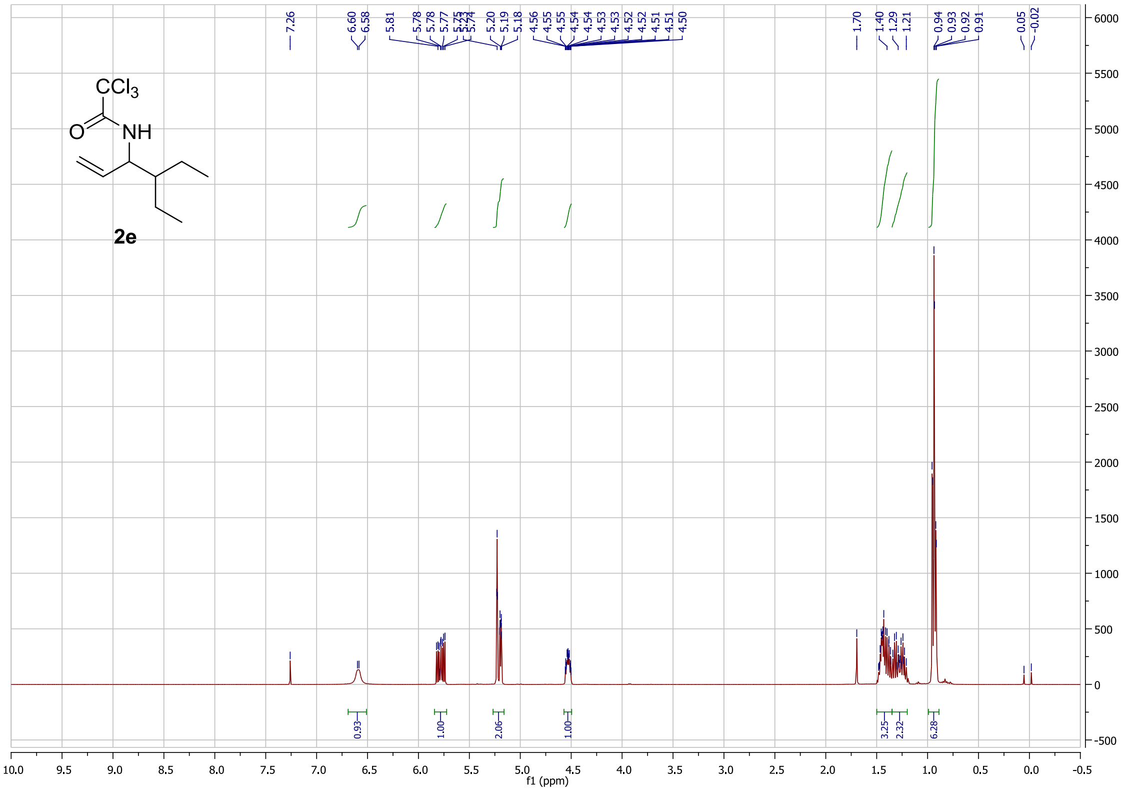

7.26
6.60
6.58
5.81
5.78
5.78
5.77
5.20
5.19
5.18
4.56
4.55
4.55
4.54
4.54
4.53
4.53
4.52
4.52
4.51
4.51
4.50
1.70
1.40
1.29
1.21
0.94
0.93
0.92
0.91
0.05
-0.02
CCl3
O
NH
2e
0.93
1.00
2.06
1.00
3.25
2.32
6.28
f1 (ppm)

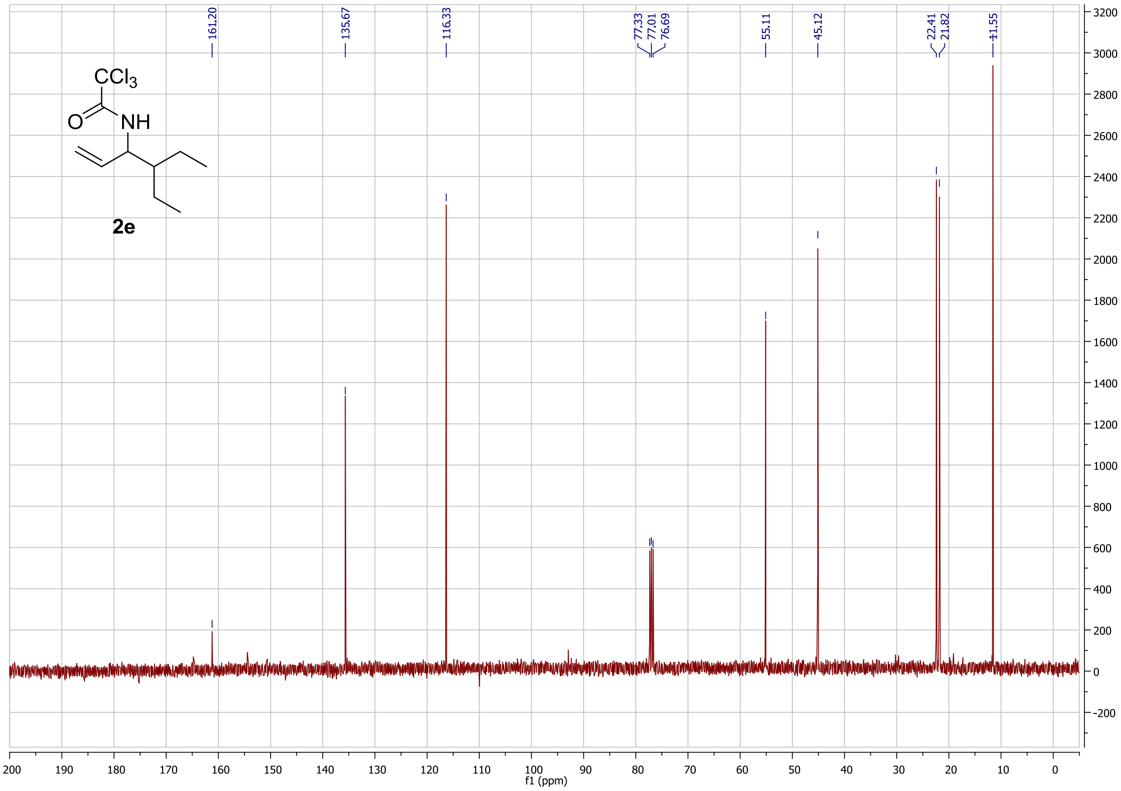

161.20
135.67
116.33
77.33
77.01
76.69
55.11
45.12
22.41
21.82
11.55
CCl3
O
NH
2e
f1 (ppm)

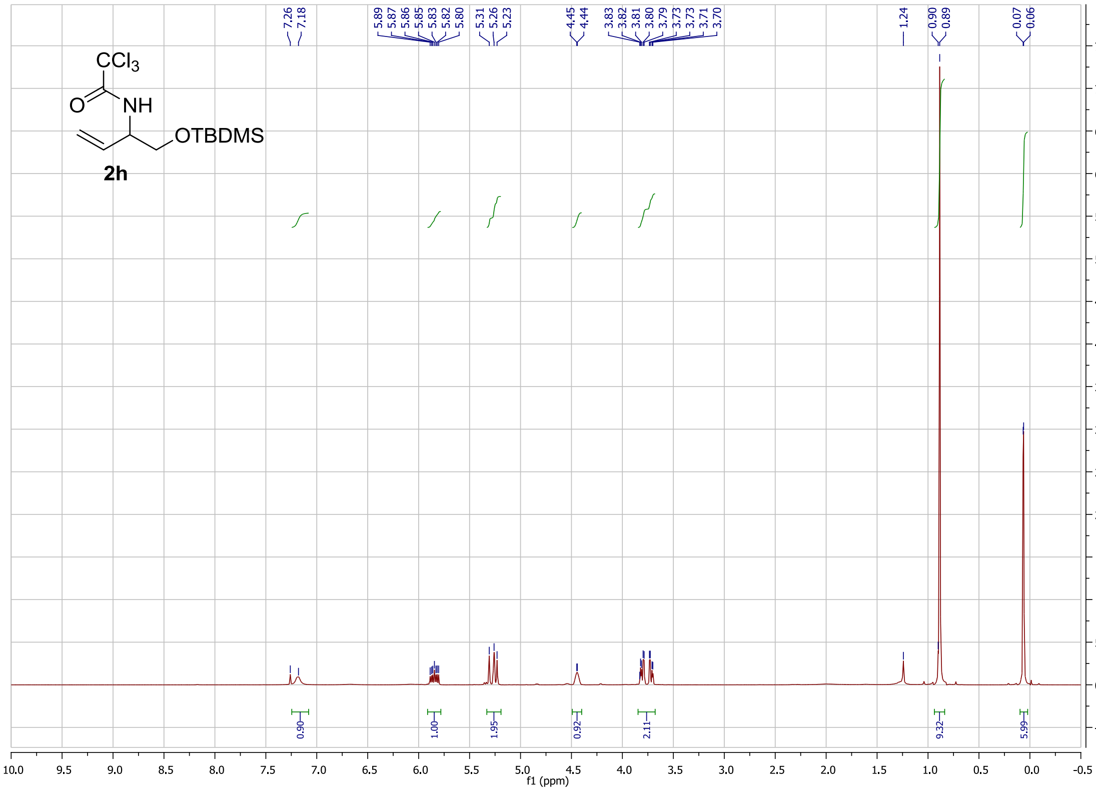
7.26
7.18
5.89
5.87
5.86
5.85
5.83
5.82
5.80
5.31
5.26
5.23
4.45
4.44
3.83
3.82
3.81
3.80
3.79
3.73
3.73
3.71
3.70
1.24
0.90
0.89
0.07
0.06
CCl3
O
NH
OTBDMS
2h
0.90
1.00
1.95
0.92
2.11
9.32
5.99
f1 (ppm)

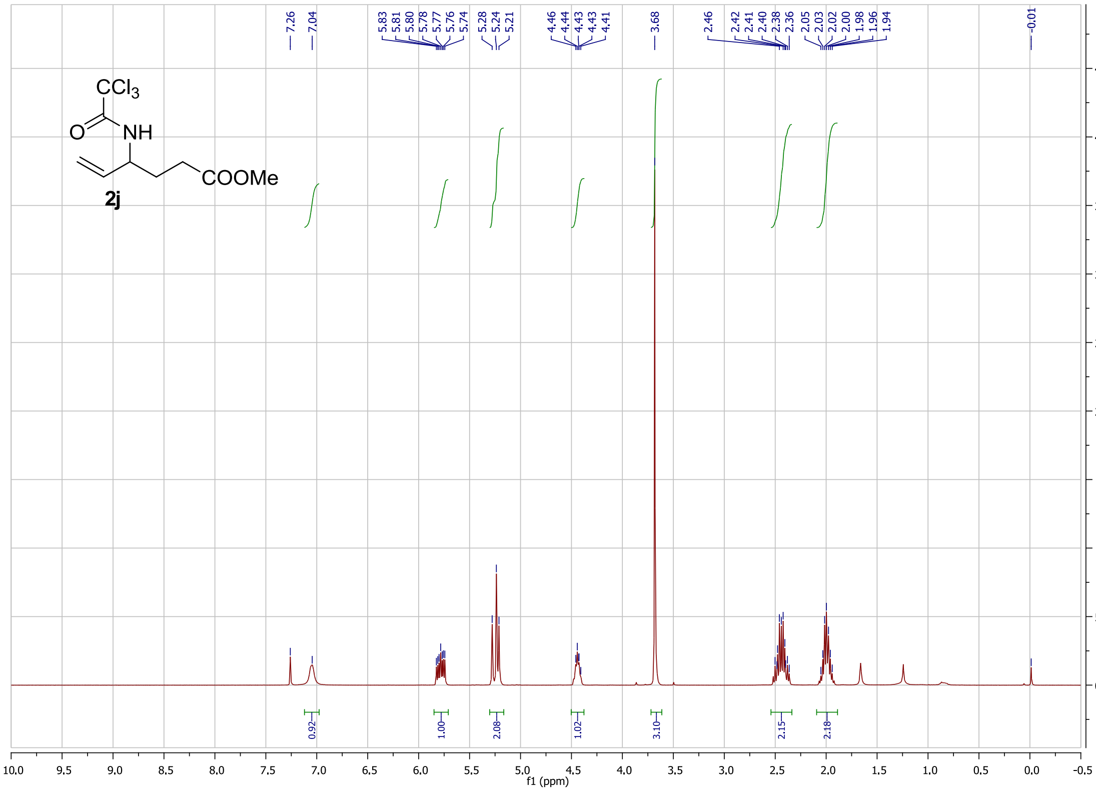
7.26
7.04
5.83
5.81
5.80
5.78
5.77
5.76
5.74
5.28
5.24
5.21
4.46
4.44
4.43
4.43
4.41
3.68
2.46
2.42
2.41
2.40
2.38
2.36
2.05
2.03
2.02
2.00
1.98
1.96
1.94
-0.01
CCl3
O
NH
COOMe
2j
0.92
1.00
2.08
1.02
3.10
2.15
2.18
f1 (ppm)

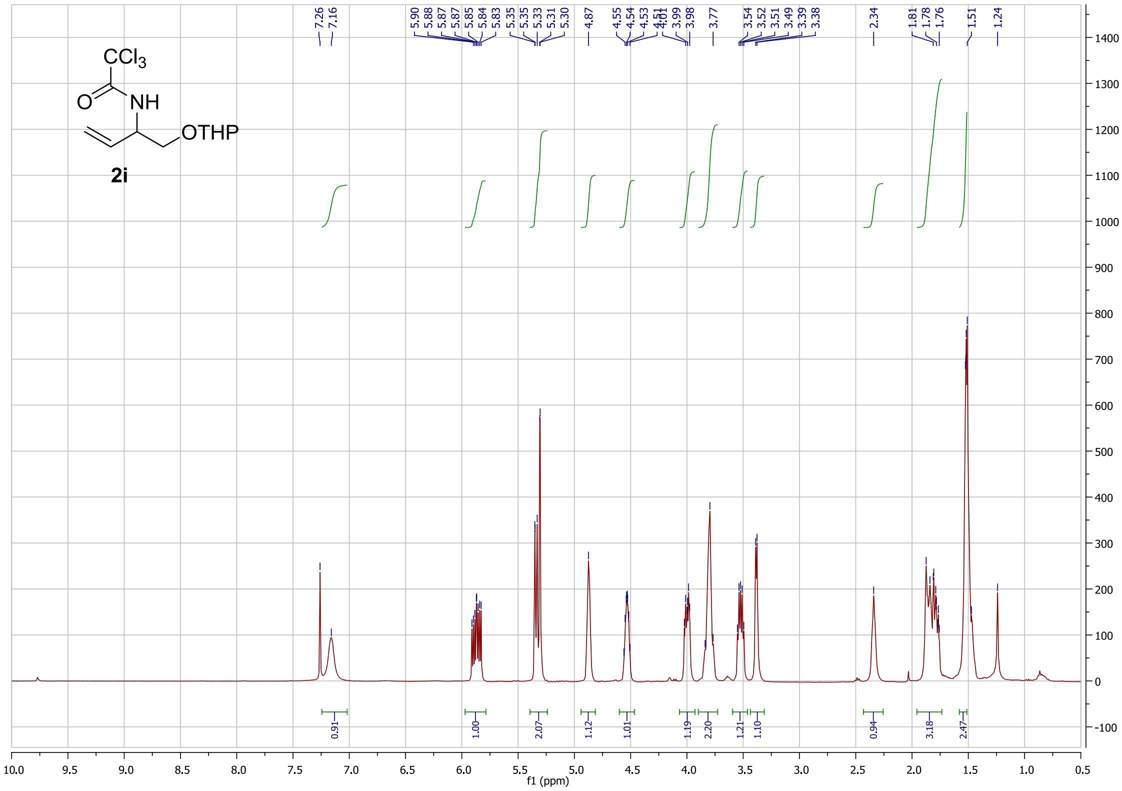
CCl3
O
NH
OTHP
2i
7.26
7.16
5.90
5.88
5.87
5.85
5.84
5.83
5.35
5.33
5.31
5.30
4.87
4.55
4.54
4.53
4.01
4.01
3.99
3.98
3.77
3.54
3.52
3.51
3.49
3.39
3.38
2.34
1.81
1.78
1.76
1.51
1.24
0.91
1.00
2.07
1.12
1.01
1.19
2.20
1.21
1.10
0.94
3.18
2.47
f1 (ppm)

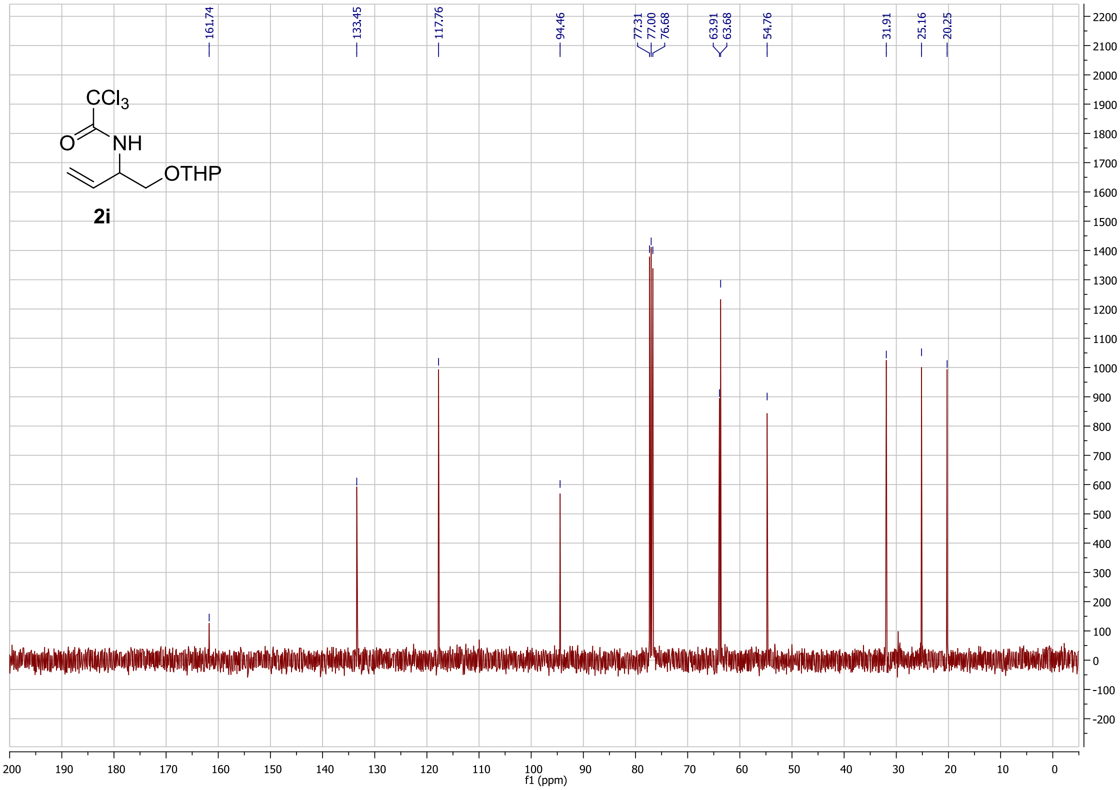
CCl3
O
NH
OTHP
2i
161.74
133.45
117.76
94.46
77.31
77.00
76.68
63.91
63.68
54.76
31.91
25.16
20.25
f1 (ppm)
